# EMB1/370: Facilitating Evidence-Based Healthcare: Clinical Practice Guidelines on the Internet

**DOI:** 10.2196/jmir.1.suppl1.e21

**Published:** 1999-09-19

**Authors:** A Bolster

**Affiliations:** ^1^Canadian Medical Association, OttawaCanada

**Keywords:** Evidence-Based Medicine, Information Systems, Internet, Practice Guidelines, User-Computer Interface

## Abstract

**Introduction:**

Evidence-based clinical practice guidelines (CPGs) can help physicians and patients make appropriate health-care decisions. However, CPGs must be readily accessible at the point of care and pertinent to the health-care environment. To facilitate access of Canadian physicians to CPGs reflecting the Canadian health care environment, the Canadian Medical Association (CMA) created the CPG Infobase (www.cma.ca/cpgs), a comprehensive, Internet-based resource.

**Methods:**

The CMA maintains a database of information on all current Canadian CPGs. Details on new CPGs and revisions, including their development, implementation and evaluation, and on related documents such as quick-reference guides for physicians and patient-oriented material, are added daily. In March 1995 the CMA began publishing online or licensing hyperlinks to evidence-based Canadian CPGs through its Web site, CMA Online, in a section named the CPG Infobase. As the CPG Infobase grew, browsing became increasingly inefficient. In addition, demand grew for information that would help the end-user determine which of several guidelines on a topic would be appropriate in a particular clinical situation. That information was available only through personal contact with the database staff. In 1998-99 the CMA developed a search engine and Web interface for the database to integrate it with the CPG Infobase. Needs assessment and iterative usability testing were conducted with physicians, the primary end-users.

**Results:**

The CPG Infobase now provides fast, one-stop access to information on more than 2000 evidence-based Canadian CPGs. By involving physicians in the design of the search engine and interface, the CMA maximized ease of use while ensuring the functionality needed by the primary end-users. Searching is flexible: novices can use a basic technique, whereas more experienced searchers can easily customize a strategy to target CPGs directly applicable to a clinical situation. The information retrieved permits the user to quickly assess which guideline is appropriate. Then, through hypertext links, the full text or structured abstract of nearly 1000 of the CPGs can be viewed, as can many of the related documents. Ordering information is available for documents not yet online.

**Discussion:**

In an era of information overload, rapid access to a thorough resource containing pertinent, reliable, evidence-based guidelines to best clinical practices is crucial for continual improvement in the quality of patient care. The Internet is an ideal access mechanism. However, acceptance and use of such a resource by physicians, who tend to be computer-search novices pressed for time, necessitate an intuitive interface and a powerful search engine. Quickly determining which of several conflicting guidelines is valid and appropriate in a specific clinical situation remains problematic. The CMA is now addressing this challenge and others related to guideline implementation.

